# An Improved Calibration Method for Photonic Mixer Device Solid-State Array Lidars Based on Electrical Analog Delay

**DOI:** 10.3390/s20247329

**Published:** 2020-12-20

**Authors:** Xuanquan Wang, Ping Song, Wuyang Zhang

**Affiliations:** Key Laboratory of Biomimetic Robots and Systems (Ministry of Education), Beijing Institute of Technology, Beijing 100081, China; 3120185108@bit.edu.cn (X.W.); 3120195107@bit.edu.cn (W.Z.)

**Keywords:** photonic mixer device, PMD solid-state array Lidar, electrical analog delay, joint calibration algorithm, self-adaptive grayscale correlation, depth calibration method

## Abstract

As a typical application of indirect-time-of-flight (ToF) technology, photonic mixer device (PMD) solid-state array Lidar has gained rapid development in recent years. With the advantages of high resolution, frame rate and accuracy, the equipment is widely used in target recognition, simultaneous localization and mapping (SLAM), industrial inspection, etc. The PMD Lidar is vulnerable to several factors such as ambient light, temperature and the target feature. To eliminate the impact of such factors, a proper calibration is needed. However, the conventional calibration methods need to change several distances in large areas, which result in low efficiency and low accuracy. To address the problems, this paper presents an improved calibration method based on electrical analog delay. The method firstly eliminates the lens distortion using a self-adaptive interpolation algorithm, meanwhile it calibrates the grayscale image using an integral time simulating based method. Then, the grayscale image is used to estimate the parameters of ambient light compensation in depth calibration. Finally, by combining four types of compensation, the method effectively improves the performance of depth calibration. Through several experiments, the proposed method is more adaptive to multiscenes with targets of different reflectivities, which significantly improves the ranging accuracy and adaptability of PMD Lidar.

## 1. Introduction

Three-dimensional information acquisition has gained extensive attention in the field of computer vision, robot navigation, human–computer interaction, automatic driving, etc. [1]. Generally, the methods to obtain three-dimensional information mainly include stereo vision [2,3], structural light [4], single-pixel 3D imaging [5] and time-of-flight (ToF) [6]. Stereo vision needs advanced matching algorithm to obtain accurate depth information, which is vulnerable to ambient light. Structural light needs projection optimization compensation, which requires high performance of the processing system. Compared with the two methods above, the ToF sensor system utilizes active infrared laser to achieve depth information acquisition, which has the advantages of low cost, high frame frequency and high reliability [7,8].

Photonic mixer device (PMD) solid-state array Lidar, as one typical kind of the ToF sensor system, is widely used in computer vision [9,10]. However, there inevitably exist several errors sources (such as ambient light, integration time, temperature drift and reflectivity), which reduce the performance of the ToF sensor significantly. Hence, the equipment needs to be properly calibrated to achieve reliable depth information acquisition [11].

Several works have been done on PMD Lidar calibration. Lindner [11,12,13,14] put forward a calibration approach, which combined the overall intrinsic, distance and reflectivity related error calibration. Compared with the other approaches, the calibration provided significant contribution to the reduction of calibration data. Kahlman [15,16] presented a parameter based calibration approach, which considered multiple error sources including integration time, reflectivity, distance and temperature. The accuracy was improved to 10 mm at the distance of 2.5 m after calibration. Steiger [17] discussed the influence of internal factors and environmental factors. Then the effect of these factors was compensated by experiments. However, the errors were above the uncertainties specified by the manufacturer even after calibration. Swadzba [18] put forward a calibration algorithm based on stepwise optimization and the particle filter framework. The experimental results showed the accuracy of the method was higher than the traditional calibration method, while the efficiency was decreased. Schiller [19] discussed a joint calibration method based on PMD camera and standard 2D CCD camera. Results showed the internal camera parameters were estimated more precisely. In addition, the limitations of the small field-of-view were overcome by the method. Fuchs [20,21] presented a calibration process for the ToF camera with respect to the intrinsic parameters, the depth measurement distortion and the pose of the camera relative to a robot’s end effector. Chiabrando [22] performed two aspects of the calibration: distance calibration and photogrammetric calibration. For distance calibration, they reduced the distance error to ±15 mm in the range of 1.5–4 m. For photogrammetric calibration, they verified the stability of the estimated camera internal parameters. Christian [23] presented a calibration approach based on the depth and reflectance image of a planar checkerboard. The method improved the efficiency and the accuracy for the calibration of the focal length and 3D pose of the camera. However, the depth accuracy was not improved. Kuhnert [24] raised a joint calibration method based on two types of 3D cameras, PMD camera and stereo camera system, to improve the range accuracy by using one camera to compensate the other one. Schmidt [25] proposed a dynamic calibration method, which can be executed on systems with limited resources. Huang [26] raised an integration time auto adaptation method based on amplitude data, which makes each pixel obtain the depth information under the best conditions. Meanwhile the Gaussian process regression model was utilized to calibrate the depth errors. He [27] analyzed the influence of several external distractions (including material, color, distance, lighting, etc.) and proposed an error correction method based on the particle filter-support vector machine (PF-SVM).

To sum up, most research on the ToF camera calibration focus on the parameters related with measurement accuracy, such as integration time, pixel related error or depth data distortion [28,29,30,31,32,33,34,35]. These methods generally need to place the calibration plate in different distances to acquire depth compensation look-up table (LUT), which require a significant amount of work. In addition, the calibration plate needs to be placed manually. Even a slight change of the angle or the location will introduce extra measuring error of several millimeters. Thus, the process is vulnerable to the human factor. Others [36,37,38,39,40,41] obtain depth compensation data by changing the attitude of ToF camera with external devices, which need complex algorithms to fuse the multisource information. Consequently, there still exist unresolved issues such as a heavy workload, complex calculation requirement and serious human disturbance. A simpler calibration method is needed to improve the applicability, accuracy and convenience of ToF cameras.

To deal with the abovementioned challenges, we [42] proposed a calibration method for PMD solid-state array Lidar based on a black-box calibration device and an electrical analog delay method in the previous work. The method solved part of the problems in traditional calibration methods, such as low efficiency, low accuracy and serious human disturbance. However, some factors still have not taken into account, such as temperature drift, targets with different reflectivity and disturbance of ambient light, which could bring extra errors during the application of PMD solid-state array Lidar.

For this reason, this paper improved the calibration setting and the method, and the main contributions are as follows:

(1) This paper proposed a self-adaptive grayscale correlation based depth calibration method (SA-GCDCM) for PMD solid-state array Lidar. Due to its special structure, the PMD Lidar has the ability to obtain a grayscale image and depth image simultaneously. Meanwhile, the amplitude of grayscale image has a close relationship with ambient light. Based on this, the grayscale image was used to estimate the parameters of ambient light compensation in depth calibration in this method. Through SA-GCDCM, the disturbance of ambient light could be effectively eliminated. Traditional joint calibration methods always need an extra RGB camera to cooperate with the ToF camera. The inconformity of the parameters of the two cameras, such as the image resolution and the field of view, can introduce extra errors to the system, leading to low calibration accuracy and efficiency. Compared with the traditional methods, this method has no requirement of coordinate transformation and feature matching, leading to better data consistency and self-adaptability.
(2) This paper proposed a grayscale calibration method based on integration time simulating. Firstly, the raw grayscale images were acquired under multiple ambient light levels through setting the integration time in several values. Then the spatial variances were calculated from the images to estimate the dark signal non-uniformity (DSNU) and photo response non-uniformity (PRNU). At last the influence of DSNU and PRNU were eliminated by a correction algorithm.
(3) Based on the electrical analog delay method, a comprehensive, multiscene adaptive multifactor calibration model was established through combining the SA-GCDCM with raw distance demodulation compensation, distance response non-uniformity (DRNU) compensation and temperature drift compensation. Compared with the prior methods, the established model is more adaptive to multiscenes with targets of different reflectivities, which significantly improves the ranging accuracy and adaptability of PMD Lidar.

The article is structured as follows: an introduction of working principle of PMD Lidar given in Section 2, along with a discussion of known error sources such as integration time, temperature and DRNU. The combined calibration method is presented in Section 3. Section 4 introduces the experiments and performs the discussion. Finally, a short summary is given in Section 5.

## 2. System Introduction

### 2.1. Principle of PMD Solid-State Array Lidar

The PMD solid-state array Lidar mainly includes three parts: the emitting unit, the receiving unit and the processing unit. The emitting unit is composed of four vertical-cavity surface-emitting laser (VCSEL), the driver, the delay-locked loop (DLL) and the modulator. Compared with the light emitting diode (LED), VCSEL has attracted attention in recent ToF lidar research [43,44,45] because of its lower power consumption, higher speed and higher reliability [46]. The receiving unit is composed of the lens, the sensor, the demodulator, the A/D converter and the sequence controller. The processing unit is a digital signal processing (DSP) controller.

**Figure 1:**
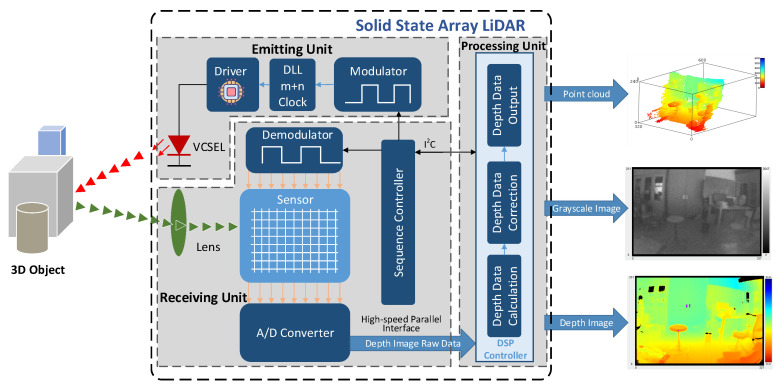
The fundamental principle of the photonic mixer device (PMD) solid-state array Lidar.

The fundamental principle [47,48] of PMD solid-state array Lidar is illustrated in Figure 1. The continuous modulated near infrared (NIR) laser is generated and emitted by the emitting unit. After reflecting at the surface of the objects, the laser is received by the receiving unit. The optical signal is converted into an electrical signal in the receiving unit. Then the electrical signal is transmitted to the processing unit, which calculates the distance data by demodulating the phase delay between the emitted and the detected signal. Finally, three types of images, point cloud, grayscale and depth images, can be output from the DSP controller through data processing.

**Figure 2:**
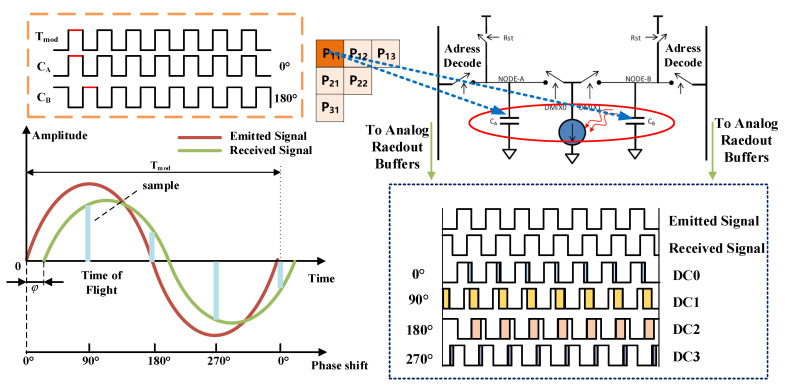
Signal demodulation method.

Signal demodulation is the key step during the working process of the PMD solid-state array Lidar, which is shown in Figure 2. Two different capacitors (C_A_ and C_B_) with two phase windows (0° and 180°) are set under each pixel of the ToF chip. The differential correlation sampling (DCS) method was used to demodulate the received signal. In general, the sampling number determined the accuracy of the demodulation, while the efficiency could be accordingly influenced. In this paper, the four-step phase-shift method was adopted for sampling. In other words, the process of demodulation was to sample the received signal at four different phases (0°, 90°, 180° and 270°) respectively by using the capacitors of two phase windows, and then suppresses the noise by obtaining the difference between these capacitors. The phase shift of the modulated light was calculated according to the sampling amplitude. At last the target distance was calculated from the phase shift.

The specific process of the four-step phase-shift method was performed as follows. The emitted light signal can be presented as E(t)=kAcos(ωt), while the received signal can be presented as R(t)=B+kAcos(ωt+Δφ). Where ω is the modulation frequency, *A* is the amplitude of the emitted signal, Δφ means the phase shift between the emitted signal and received signal, *B* is the noise signal generated during the transmission of light and *k* means the signal attenuation coefficient. The sampling process can be expressed in Equation (1):

(1) $$\begin{array}{ll} Q_{1}^{DC0}=\int_{0}^{\frac{\pi }{\omega }}\left[B+kAcos(\omega t+\Delta \varphi )\right]dt & Q_{2}^{DC0}=\int_{\frac{\pi }{\omega }}^{\frac{2\pi }{\omega }}\left[B+kAcos(\omega t+\Delta \varphi )\right]dt\\ Q_{1}^{DC1}=\int_{\frac{\pi }{2\omega }}^{\frac{3\pi }{2\omega }}\left[B+kAcos(\omega t+\Delta \varphi )\right]dt & Q_{2}^{DC1}=\int_{\frac{3\pi }{2\omega }}^{\frac{5\pi }{2\omega }}\left[B+kAcos(\omega t+\Delta \varphi )\right]dt\\ Q_{1}^{DC2}=\int_{\frac{\pi }{\omega }}^{\frac{2\pi }{\omega }}\left[B+kAcos(\omega t+\Delta \varphi )\right]dt & Q_{2}^{DC2}=\int_{\frac{2\pi }{\omega }}^{\frac{3\pi }{\omega }}\left[B+kAcos(\omega t+\Delta \varphi )\right]dt\\ Q_{1}^{DC3}=\int_{\frac{3\pi }{2\omega }}^{\frac{5\pi }{2\omega }}\left[B+kAcos(\omega t+\Delta \varphi )\right]dt & Q_{2}^{DC3}=\int_{\frac{5\pi }{2\omega }}^{\frac{7\pi }{2\omega }}\left[B+kAcos(\omega t+\Delta \varphi )\right]dt \end{array}$$

where $Q_{1}^{DC_{i}}$ and $Q_{2}^{DC_{i}}$, are the integral values of capacitors *C_A_* and *C_B_* at sampling point *i*, respectively.

(2) $$\begin{array}{ll} DC0=Q_{1}^{DC0}-Q_{2}^{DC0} & DC1=Q_{1}^{DC1}-Q_{2}^{DC1}\\ DC2=Q_{1}^{DC2}-Q_{2}^{DC2} & DC3=Q_{1}^{DC3}-Q_{2}^{DC3} \end{array}$$

The distance is calculated by Equation (3):

(3) $$D_{raw}\left(x,y\right)=\frac{c}{2}\times \frac{1}{2\pi f}\times atan2\left(\frac{DC3\left(x,y\right)-DC1\left(x,y\right)}{DC2\left(x,y\right)-DC0\left(x,y\right)}\right)$$

### 2.2. Analysis of Error Sources of PMD Solid-State Array Lidar

The PMD Lidar is vulnerable to several factors such as internal non-uniformity of the ToF sensor, demodulation process, temperature drift, ambient light, etc. Some of the factors have been discussed in our previous work [42], which will not be mentioned in this paper. However, factors like integration time, temperature drift and DRNU still need to be considered. The analysis of these factors and the qualitative study are described in detail as follows.

#### 2.2.1. Integration Time

**Figure 3:**
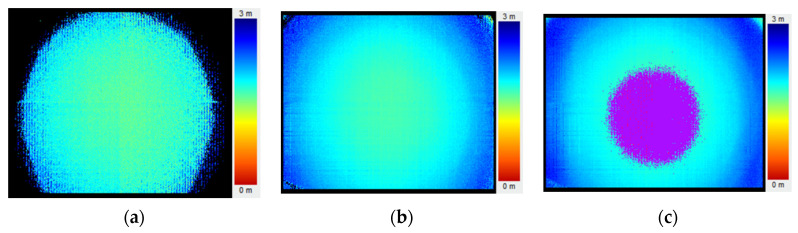
Depth images under different integration time. (**a**) under 50 μs; (**b**) under 300 μs and (**c**) under 650 μs. The images were captured with a flat board.

Integration time is the time span to output individual data. In general, the integration time can be set from tens to thousands of microseconds. Too short integration time brings the loss of local information, as shown in Figure 3a. While too long integration time will exceed the ToF sensor’s receiving range, leading to a local saturation, as shown in Figure 3c. A proper value is needed to capture a sufficient number of photoelectrons without saturation, as shown in Figure 3b.

#### 2.2.2. Temperature Drift

**Figure 4:**
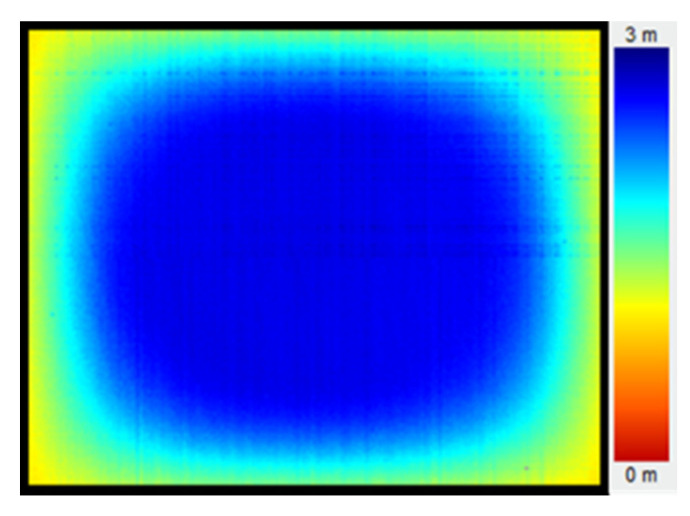
Non-uniform measurement due to uneven temperature distribution.

The ToF sensor is susceptible to environment temperature and the heat generated by itself during its working, leading to an uneven temperature distribution. However, the electron mobility in the sensor is temperature dependent. The higher the temperature, the lower the electron mobility, leading to a non-uniformity of measurement, as shown in Figure 4.

In addition, the illumination driver and the external circuit also have temperature dependent demodulation delay, which affect the distance measurement.

#### 2.2.3. Distance Response Non-Uniformity (DRNU)

The ToF sensor typically have many analog to digital converters (ADCs) arranged along the columns of the pixel-field. The different ADCs have slightly different behaviors and result in a non-uniformity between the columns. In addition, this type of error exists in row and due to the non-uniformity of the row addressing signals. These two types of non-uniformities lead to differences of demodulation from pixel to pixel, which is called distance response non-uniformity (DRNU). This type of error also needs to be compensated.

For instance, the phase shift is calculated with Equation (4):

(4) $$\varphi =atan\left(\frac{DC3-DC1}{DC2-DC0}\right)$$

While the real phase shift without DRNU compensation is calculated with Equation (5):

(5) $$\varphi =atan\left(\frac{\left(DC3+a)-(DC1+b\right)}{\left(DC2+c)-(DC0+d\right)}\right)$$

**Figure 5:**
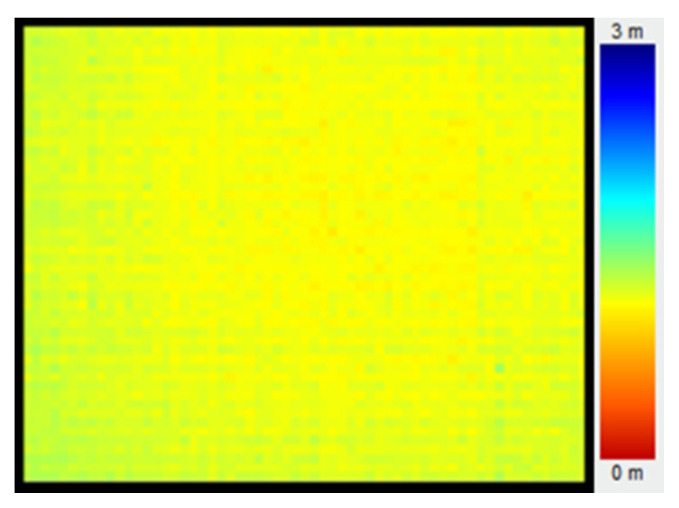
Depth image without distance response non-uniformity (DRNU) compensation.

Figure 5 demonstrates the depth image without DRNU compensation, where the non-uniformity was obviously unneglectable.

## 3. Methodology

**Figure 6:**
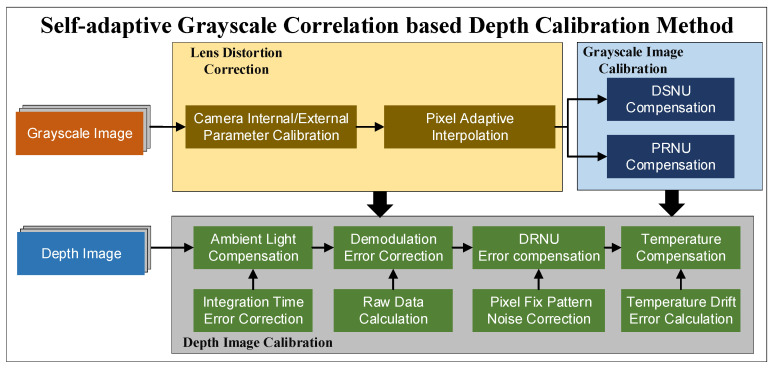
The comprehensive calibration model process.

The conventional calibration methods are time-consuming and complex due to the existence of multiple error sources. Based on the previous work, this paper put forward an improved calibration method based on the electrical analog delay. The method fused various error compensations into a comprehensive calibration model, where the grayscale image and the depth image were calibrated jointly, as shown in Figure 6. The lens distortion was corrected using a self-adaptive interpolation algorithm based on Zhang’s [49] calibration method. For grayscale image correction, DSNU and PRNU were compensated based on an integration time simulating based method. For depth information correction, the grayscale image was used to estimate the parameters of ambient light compensation. After calculating the raw depth data, the pixel fix pattern noise was eliminated by DRNU error compensation. The temperature drift error was compensated at last.

### 3.1. Lens Distortion Correction

The internal and external parameters of PMD solid-state array Lidar were obtained through Zhang’s [49] calibration method, which is not detailed in this paper. Different from the grayscale image, there may exist the holes on the depth image (the received signal is too low to demodulate a valid signal), resulting in the inapplicability of the traditional correction algorithm. A pixel adaptive interpolation strategy we proposed in [42] was utilized in this paper to solve the problem, which is presented in Table 1.

Green points represent the projection of the corrected pixels on the raw image. Purple points are the surrounding pixels of the green ones, in which the solid purples mean the real distance pixel while the hollow ones represent the holes. *D_0_*, *D_1_*, *D_2_*, *D_3_* and *D_4_* denote the pixels to be interpolated and its lower-left pixel, top-left pixel, lower-right pixel and top-right pixel, respectively. (*x*_p0_,*y*_p0_), (*x*_p1_,*y*_p1_), (*x*_p2_,*y*_p2_), (*x*_p3_,*y*_p3_) and (*x*_p4_,*y*_p4_) are the coordinates of the pixels to be interpolated and its lower-left pixel, top-left pixel, lower-right pixel and top-right pixel, respectively. *α_x_* = *x_p_*_0_ − *x_p_*_1_, *α_y_* = *y_p_*_0_ − *y_p_*_1_ and (*u*,*v*) are the coordinates of the pixels to be interpolated under a barycentric coordinate system.

### 3.2. Grayscale Image Calibration

A grayscale image is acquired by TOF chip when PMD solid-state Array Lidar works in the passive mode. The acquisition process is consistent with the intensity image obtained by traditional complementary metal oxide semiconductor (CMOS) chip, which can be used to characterize the intensity of ambient light.

In general, the grayscale image is vulnerable to DSNU and PRNU, where DSNU represents the differences of gray values between pixels captured under the dark condition and PRNU represents the differences of gray values between pixels captured under the common condition, respectively.

The most commonly used approach to correct the influence of DSNU and PRNU has a standardized process, which can be found in european machine vision association (EMVA) standard 1288 [50]. In this approach, one dark image and one bright image are captured under the same exposure condition. DSNU and PRNU are then calculated from the images. However, there exist some limitations of the approach. For instance, the images are captured under a specific condition, leading to bad applicability. The ambient light is set artificially, which introduces extra error. Based on this approach, an integration time simulating based method was proposed in this paper. The main contributions of the method mainly include: (I) Instead of setting the ambient light artificially, the levels of ambient light were simulated by setting the integration times and (II) the non-uniform of the exposure was eliminated by calculating the mean value of multiple ambient light levels. The process of the method is given as follows.

(1) Set the integration time to 0 μs to simulate the dark condition. Collect *N* = 100 frames of grayscale images and calculate the mean value.
  (6) $$Y_{dark-avg}=\frac{1}{N}\sum_{x=1}^{320}\sum_{y=1}^{240}{\left(x,y,N\right)}_{dark}/\left(320\times 240\right)$$
(2) Change the integration time to simulate different ambient light levels. Collect *N* = 100 frames of grayscale images under amplitudes of 10%, 30%, 50% and 80% respectively. Similarly, the mean values with different amplitudes are obtained.
  (7) $$Y_{dark-avg}=\frac{1}{N}\sum_{x=1}^{320}\sum_{y=1}^{240}{\left(x,y,N\right)}_{AL}/\left(320\times 240\right)$$
(3) Calculate the spatial variances under different ambient levels. Spatial variance is simply an overall measure of the spatial nonuniformity, which is helpful to estimate DSNU and PRNU.
  (8) $$S_{dark}^{2}={\sum_{x=1}^{320}\sum_{y=1}^{240}\left[{\left(x,y\right)}_{dark}-Y_{dark-avg}\right]}^{2}/\left(320\times 240-1\right)\;S_{AL}^{2}={\sum_{x=1}^{320}\sum_{y=1}^{240}\left[{\left(x,y\right)}_{AL}-Y_{AL-avg}\right]}^{2}/\left(320\times 240-1\right)$$
(4) Calculate the correction values of DSNU and PRNU.
  (9) $$b_{DSNU}=S_{dark}\;k_{PRNU}=\sqrt{S_{{}_{AL}}^{2}-S_{{}_{dark}}^{2}}/\left(Y_{AL-avg}-Y_{dark-avg}\right)$$
(5) The grayscale compensation of pixel (x,y) is calculated by Equation (10).
  (10) $$I_{corr}\left(x,y\right)=\left[I_{raw}\left(x,y\right)-b_{DSNU}\left(x,y\right)\right]\times k_{PSNU}$$
  where $Y_{dark-avg}$ is the mean value of grayscale images under the dark condition, $Y_{AL-avg}$ is the mean value of grayscale images under ambient light, *N* means the number of frames, $S_{{}_{dark}}^{2}$ and $S_{{}_{AL}}^{2}$ are spatial variances under dark and ambient light conditions, respectively, $b_{DSNU}$ is the offset of DSNU, $k_{PRNU}$ is the gain of PRNU and $I_{corr}\left(x,y\right)$ is the compensation value of grayscale image after calibration.

### 3.3. Depth Image Calibration

#### 3.3.1. Ambient Light Compensation

Due to its special structure, the PMD solid-state array Lidar has the ability to obtain a grayscale image and depth image simultaneously. Meanwhile, the amplitude of a grayscale image has a close relationship with ambient light. Based on this, the grayscale image was used to estimate the parameters of ambient light compensation in depth calibration, which is the self-adaptive grayscale correlation based depth calibration method (SA-GCDCM). The basic idea of the method is to eliminate the influence of ambient light in the sampling stage by introducing an ambient light correction factor *K_AL_*. The factor *K_AL_* is calculated from several *DCs* sampled under different ambient light levels. The ambient light is controlled accurately by adjusting the integration time. *K_AL_* is then utilized to correct the *DCs* in the real sampling process. There exists internal noise and external error during the sampling. The errors are corrected in the calculation. The process of the method is concluded as (all the following measurements use the spatial average of the region of interest (ROI) within the coordinates (100,70) and (220,165) and the temporal average of 100 frames as a default):

(1) Turn the ambient light on and record the amplitude of the grayscale image as *Q*_gray_.
(2) Change the amplitude of the grayscale image to 0.5 times that of *Q*_gray_ by adjusting the integration time. Measure the *DC0/2* and record as *DC0_setting1_* and *DC2_setting1_*, respectively.
(3) Change the amplitude of the grayscale image to 1.5 times that of *Q*_gray_ by adjusting the integration time. Measure the *DC0/2* and record as *DC0_setting2_* and *DC2_setting2_*, respectively.
(4) Turn the ambient light off and measure the *DC0/2*, which are recorded as *DC0_no_* and *DC2_no_*, respectively.
(5) Calculate four measurements, *Q0_1_*, *Q0_2_*, *Q2_1_* and *Q2_2_*.
  (11) $$\begin{array}{ll} Q0_{1}=DC0_{setting1}-DC0_{no} & Q2_{1}=DC2_{setting1}-DC2_{no}\\ Q0_{2}=DC0_{setting2}-DC0_{no} & Q2_{2}=DC2_{setting2}-DC2_{no} \end{array}$$
(6) Correct the errors generated in the sampling. There inevitably exists internal noise and external error. The internal noise mainly comes from the internal circuit and can be eliminated by subtracting two samples at the same phase. The external error mainly comes from the instability of the environment. It can be suppressed by calculating the mean value of the samples.
  (12) $$k=\frac{\left(Q2_{2}-Q2_{1}\right)+\left(Q0_{2}-Q0_{1}\right)}{2}$$
(7) The ambient light correction factor KAL is calculated by Equation (13):
  (13) $$K_{AL}=\frac{Q_{gray}}{k}$$
  where *DC0/1/2/3* are the sample values acquired at 0°, 90°, 180° and 270° respectively. *K_AL_* is used to compensate the ambient light error during the demodulation process as Equation (14):
  (14) $$DC0/1_{corr}\left(x,y\right)=DC0/1\left(x,y\right)-\frac{I_{corr}\left(x,y\right)}{K_{AL}}$$
  where $DC0/1_{corr}\left(x,y\right)$ represents the corrected value of DC0/1(x,y).

As introduced in Section 2.1, distance measurements are taken by acquiring the four *DCs* and calculated pixel-by-pixel during runtime as Equation (15):

(15) $$D_{raw}\left(x,y\right)=\frac{c}{2}\times \frac{1}{2\pi f}\times atan2\left(\frac{DC3\left(x,y\right)-DC1\left(x,y\right)}{DC2\left(x,y\right)-DC0\left(x,y\right)}\right)$$

The equation is revised after ambient light compensation as Equation (16):

(16) $$D_{raw}\left(x,y\right)=\frac{c}{2}\times \frac{1}{2\pi f}\times atan2\left(\frac{DC3\left(x,y\right)-DC1_{corr}\left(x,y\right)}{DC2\left(x,y\right)-DC0_{corr}\left(x,y\right)}\right)$$

where $DC1_{corr}\left(x,y\right)$ and $DC0_{corr}\left(x,y\right)$ are sample values corrected by $K_{AL}$.

Through SA-GCDCM, the disturbance of ambient light could be effectively eliminated. Compared with the traditional joint method using a common RGB camera with a ToF camera, this method has no requirement of coordinate transformation and feature matching, leading to a better data consistency and self-adaptability.

#### 3.3.2. Demodulation Error Correction

**Figure 7:**
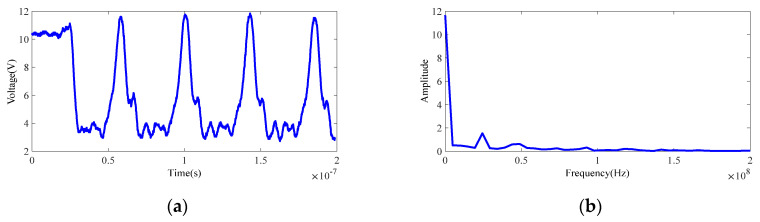
The actual sinusoidal modulation signal and its Fourier transform. (**a**) Actual sinusoidal modulation signal. (**b**) Fast Fourier transform of the sinusoidal modulation signal.

In general, the sinusoidal wave is adopted as the modulated continuous wave signal, which can be represented as E(t)=kAcos(ωt). Similarly, the received signal is deemed as a sinusoidal wave in demodulation as well. However, because of the limitations of the generator bandwidth, the actual received signal is similar to a rectangular wave [42], as shown in Figure 7. Therefore, the rectangular wave was used for demodulation analysis in this paper.

Different from Section 2.1, the sampling process is modified as Equation (17):

(17) $$\begin{array}{ll} Q_{1}^{DC0}=At_{ToF}\;\;\;\;0<t_{ToF}<\frac{\pi }{w} & Q_{2}^{DC0}=A(\frac{\pi }{w}-t_{ToF})\;\;\;\;0<t_{ToF}<\frac{\pi }{w}\\ \left\{ \begin{array}{ll} Q_{1}^{DC1}=A(\frac{\pi }{2w}-t_{ToF}) & 0<t_{ToF}<\frac{\pi }{2w}\;\\ Q_{1}^{DC1}=A(t_{ToF}-\frac{\pi }{2w}) & \frac{\pi }{2w}<t_{ToF}<\frac{\pi }{w} \end{array}\right. & \left\{ \begin{array}{ll} Q_{2}^{DC1}=A(t_{ToF}+\frac{\pi }{2w}) & 0<t_{ToF}<\frac{\pi }{2w}\;\\ Q_{2}^{DC1}=A(\frac{3\pi }{2w}-t_{ToF}) & \frac{\pi }{2w}<t_{ToF}<\frac{\pi }{w} \end{array}\right.\\ Q_{1}^{DC2}=A(\frac{\pi }{w}-t_{ToF})\;\;\;\;0<t_{ToF}<\frac{\pi }{w} & Q_{2}^{DC2}=At_{ToF}\;\;\;\;0<t_{ToF}<\frac{\pi }{w}\\ \left\{ \begin{array}{ll} Q_{1}^{DC3}=A(t_{ToF}+\frac{\pi }{2w}) & 0<t_{ToF}<\frac{\pi }{2w}\;\\ Q_{1}^{DC3}=A(\frac{3\pi }{2w}-t_{ToF}) & \frac{\pi }{2w}<t_{ToF}<\frac{\pi }{w} \end{array}\right. & \left\{ \begin{array}{ll} Q_{2}^{DC3}=A(\frac{\pi }{2w}-t_{ToF}) & 0<t_{ToF}<\frac{\pi }{2w}\;\\ Q_{2}^{DC3}=A(t_{ToF}-\frac{\pi }{2w}) & \frac{\pi }{2w}<t_{ToF}<\frac{\pi }{w} \end{array}\right. \end{array}$$

Thus, an extra error, which is called the demodulation error is generated in the revising of the sampling process. The method to correct demodulation error has been discussed extensively in [42], which will not be introduced in this paper.

#### 3.3.3. DRNU Error Compensation

The calibration was based on the electrical analog delay method. Instead of changing the real distance, delay-locked loop was used to simulate the distance in this method. The simulated distance is composed of two parts, as shown in Equation (18). The first part is the simulated distance of DLLs. The system contains several DLLs and each DLL represents a specific simulated distance, e.g., 0.3 m. The second part represents the real distance between the calibration plate and the PMD Lidar. Through combining the two parts, multiple distances could be simulated without moving the calibration plate. However, DLL is susceptible to temperature changing and circuit delay, leading to a deviation between simulated distance and the set distance. Thus, a DRNU error compensation was conducted as Equation (19):

(18) $$D_{sim}(x,y)=n\times d_{DLL}+o_{zero}$$

(19) $$DRNU(x,y)=D_{cal}(x,y)-D_{sim}(x,y)$$

where *d_DLL_* represents the simulated distance of a single DLL, *n* is the number of DLLs, *o_zero_* is the distance between the PMD solid-state array Lidar and the reflecting plate, *D_sim_(x,y)* represents the overall simulated distance, *D_cal_(x,y)* represents the corrected distance after compensation and *DRNU(x,y)* is the compensation value.

Since the DRNU error is related with distance, and the limited number of compensate values cannot completely cover the whole distance. A linear interpolation was carried out to obtain a continuous offset curve. The interpolate method is quite basic and will not be illustrated here.

#### 3.3.4. Temperature Compensation

Several research have reported the influence of the temperature for the ToF camera [7,15,35]. In this paper, the main components related to temperature error in the PMD Lidar were further classified into three parts, which are the ToF sensor, the illumination driver and the external circuit, as discussed in Section 2.2.2. It was found from experiments that the error showed a linear relation with temperature, from which the higher the temperature, the higher the error was. Since the error arose from temperature drift was compensated with a joint equation, as shown in Equation (20):

(20) $$D_{final}\left(x,y\right)=D_{cal}\left(x,y\right)-\left(T_{act}-T_{cal}\right)\times \left(TC_{pix}+TC_{laser}+n\times TC_{DLL}\right)$$

where *D_final_(x,y)* is the corrected distance after temperature compensation, *T_act_* represents the acting temperature, *T_ca_*_l_ means the temperature during the calibration, *TC_pix_* is the temperature coefficient of the pixel, *TC_laser_* means the temperature coefficient of the illumination unit and *TC_DLL_* represents the temperature coefficient of DLL stage.

For the device in this paper, the *TC_pix_* was 11.3 mm/K, the *TC_laser_* was 2.7 mm/K and the *TC_DLL_* was 0.7 mm/K. It is worth mentioning that the parameters were obtained by the specific device, which means the parameters are not applicable for each device. This is due to the diversity of the circuit board, chip and other components derived from fabrication.

After utilizing the temperature compensation, the multiscene adaptive multifactor calibration model was established. Then the feasibility of the model was verified by experiments in Section 4.

## 4. Experiments and Discussions

### 4.1. Experimental Settings

**Figure 8:**
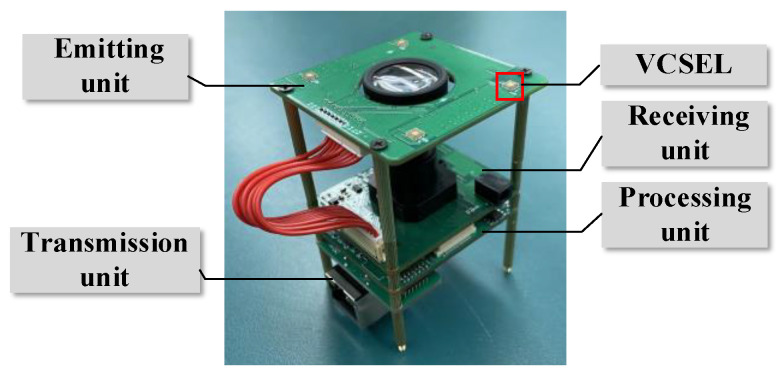
The PMD solid-state array Lidar.

The PMD solid-state array Lidar is shown in Figure 8, which is mainly composed of four parts: the emitting unit, the receiving unit, the processing unit and the transmission unit. The emitting unit generates and emits the NIR light with the VCSEL. The receiving unit receives the returned light with a CMOS sensor and converts the optical signal into an electrical signal. The processing unit calculates the distance data by demodulating the phase delay between the emitted and the detected signal. The transmission unit transmits the distance data to the computer.

**Figure 9:**
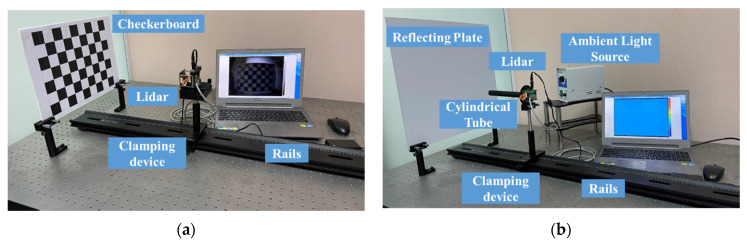
Experimental settings. (**a**) The grayscale image calibration system and (**b**) the depth image calibration system.

As illustrated in Figure 9, the experimental settings were established. The grayscale image calibration system, as shown in Figure 9a, was used to calibrate the lens distortion and eliminate the effect of DSNU and PRNU. The depth image calibration system, as shown in Figure 9b, was used to compensate multierror sources in distance measurements.

The grayscale image calibration system mainly includes the checkerboard, the PMD solid-state array Lidar, the clamping device and the rail. Based on the system, the grayscale image calibration was conducted as follows.

(1) Clamp the PMD Lidar on the clamping device.
(2) Adjust the clamping device to a proper location where the checkerboard is suitable in size and position in the field of view of the PMD Lidar.
(3) Calibrate the grayscale image based on integration time simulating.
(4) Obtain several grayscale images with checkerboard in different directions to calibrate the lens distortion.
(5) Utilize the pixel adaptive interpolation strategy to fill the holes.

The depth image calibration system mainly includes the reflecting plate, the PMD solid-state array Lidar, the cylindrical tube, the ambient light source, the clamping device and the rail. The cylindrical tube was used to protect the ToF sensor from affecting the stray light. The ambient light source was used to provide the assistant lighting. Based on the system, the depth image calibration was conducted as follows.

(1) Install the cylinder on the PMD Lidar and clamp the PMD Lidar on the clamping device.
(2) Adjust the clamping device to a proper location where the quality of the light spot projected on the reflecting plate is optimized.
(3) Change the distance with the electrical analog delay method to perform the depth calibration. Multiple error compensation is included in this step.
(4) Change the reflecting board to adjust the method with objects of different reflectivities.
(5) Conduct the interpolation on the data to obtain the continuous offset curves.

### 4.2. Results with Grayscale Image Calibration

#### 4.2.1. Lens Distortion Correction

**Figure 10:**
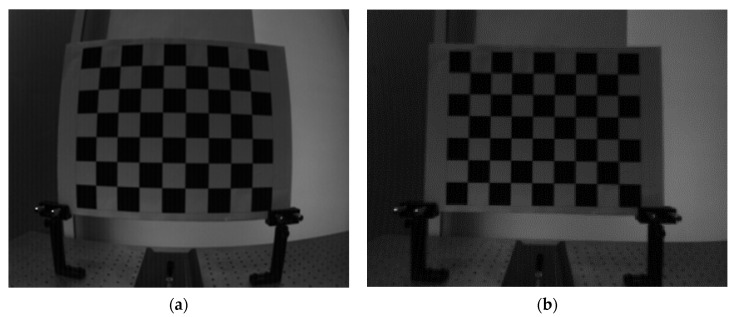
Results of lens distortion correction. (**a**) Before calibration and (**b**) after calibration.

Several grayscale images with checkerboard in different directions were obtained to calibrate the lens distortion. The pixel adaptive interpolation strategy was used to fill the holes. The results are shown Figure 10.

The internal parameters and the distortion coefficients are shown in Table 2.

#### 4.2.2. DSNU and PRNU

The influences of DSNU and PRNU were eliminated based on the integration time simulating method introduced in Section 3.2. Firstly, the raw grayscale images were acquired under multiple ambient light levels through setting the integration time in several values. Then the spatial variances were calculated from the images to estimate the dark signal non-uniformity (DSNU) and photo response non-uniformity (PRNU). At last the influence of DSNU and PRNU were eliminated by a correction algorithm.

**Figure 11:**
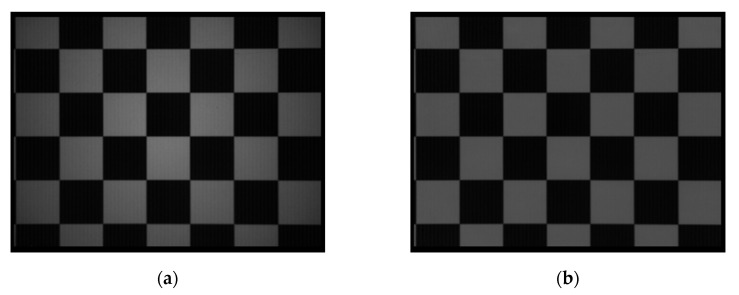

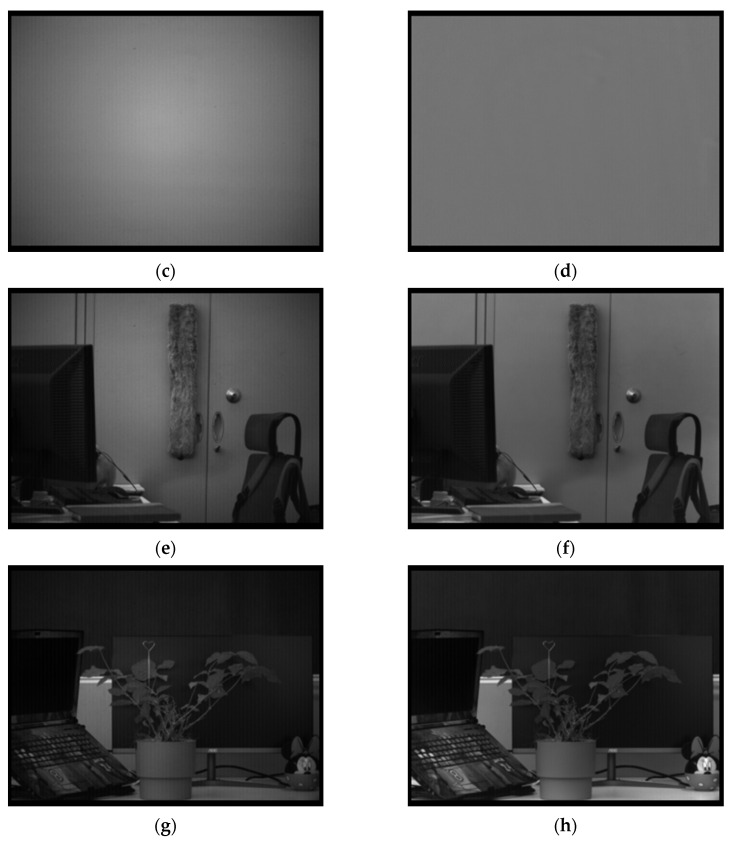
Results of grayscale image calibration. Images (**a**, **c**, **e**, **g**) are captured before calibration, while images (**b**, **d**, **f**, **h**) are captured after calibration.

To evaluate the effectiveness of the method, several experiments were conducted in different scenes. A checkerboard and a flat white board were used as the test scenes to conduct a qualitative analysis and a quantitative analysis. Then gray images were captured under two real scenes to verify the feasibility of the method. The results are shown in Figure 11.

The images in the left column were captured before calibration, while the images in the right column were captured after calibration. Figure 11a,b were captured with the checkerboard. Compared with the image before calibration, two types of non-uniformity were compensated. In the raw image, the central area was brighter while the surroundings were darker because of the uneven exposure. Meanwhile, there existed distinct light and dark stripes in vertical. In the images after calibration, these two phenomena were suppressed obviously.

To better prove the effectiveness of the method, a quantitative analysis was then conducted. A flat white board was suitable to conduct the analysis because of its good flatness and smoothness. The images are shown in Figure 11c,d. The improvement in visual was consistent with results of the checkerboard. Two types of non-uniformities were compensated obviously. To better verify the improvement, mean value, root mean square error (RMSE) and peak signal to noise ratio (PSNR) [51] were chosen to evaluate the quality of the images, and the results are shown in Table 3. The mean value shows little difference before and after the calibration, which means the method did not change the overall sampling of the grayscale signal. However, the RMSE got a significant reduction after calibration, which indicates the uniformity of the grayscale signal was improved distinctly. In addition, the PSNR improved after calibration, which means the noise derived from PRNU and DSNU was reduced.

Experiments were then conducted in two real scenes to verify the applicability of the method in reality, as shown in Figure 11e–h. It can be seen that the vertical stripes were effectively suppressed after calibration. Meanwhile, the uneven exposure, which leads to uneven brightness of the image, was obviously suppressed. Similarly, quantitative analysis was conducted and the results are shown in Table 3. The mean values showed no distinct differences before and after the calibration, while the reduction of the RMSEs was obvious. It means that the non-uniformity of the grayscale images was suppressed. The PSNRs were higher after calibration, which means the noise was effectively reduced. The results in real scenes were in accordance with results in test scenes, which mean the calibration method is feasible in reality.

### 4.3. Result with Depth Image Calibration

**Figure 12:**
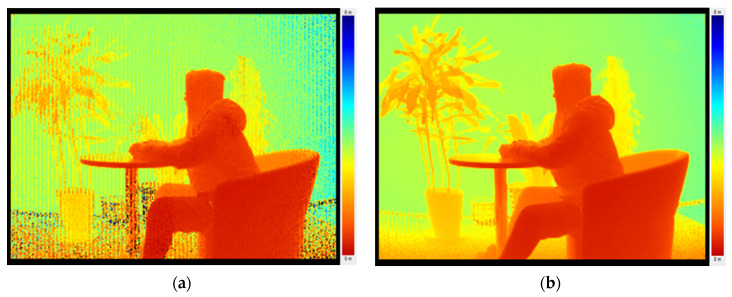
Results of depth image calibration. (**a**) Before calibration and (**b**) after calibration.

Depth image calibration was carried out after grayscale image calibration. The results are shown in Figure 12. In the left image, which was obtained before calibration, there existed many incorrect even invalid data points, while the bright and dark stripes can be observed distinctly. The non-uniformity of the whole image indicates the depth data was untrustworthy before calibration. After utilizing ambient light compensation, demodulation error correction, DRNU error compensation and temperature compensation, the quality of the depth image improved significantly. The number of noise points reduced obviously. The confidence of depth information was greatly improved.

### 4.4. Ranging Accuracy Verification under Real Environment

**Figure 13:**
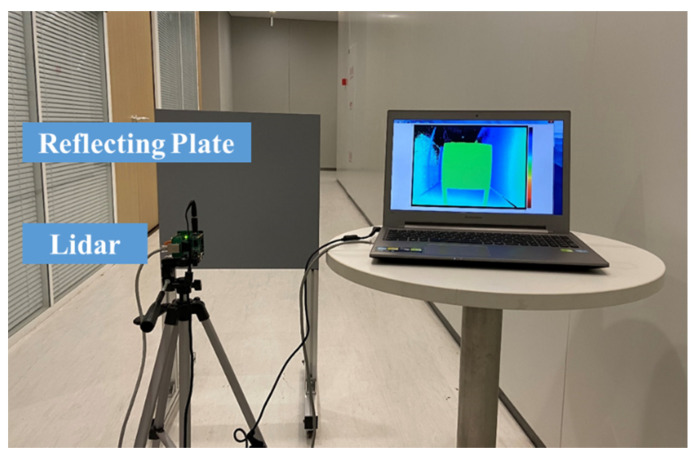
The test system.

To verify the ranging accuracy and the adaptability of the calibration method, several tests under the real environment were conducted. The test system is shown in Figure 13. Firstly, the test system was placed in the dark environment (the ambient light was about 0 Lux), indoor environment (about 500 Lux) and outdoor environment (about 1200 Lux), respectively. Then the reflecting plate was set as 80% reflectivity, 50% reflectivity and 20% reflectivity, respectively.

**Figure 14:**
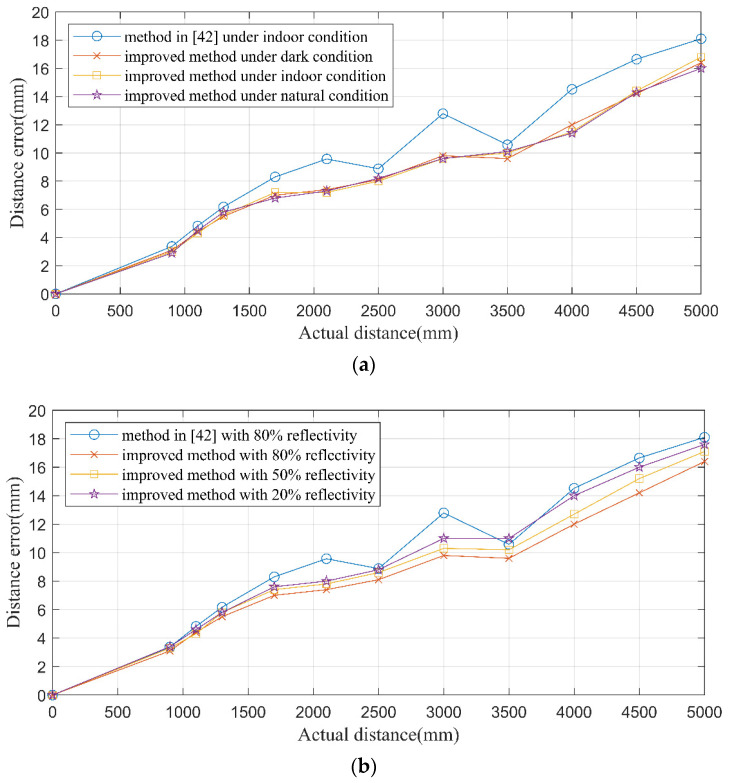
The test results. (**a**) Under different ambient conditions and (**b**) with targets of different reflectivities.

In each test, the distance between the PMD solid-state array Lidar and the reflecting plate changed from 0.5 to 5 m in a gradient. The mean value in ROI (1000 pixels in the central region) was recorded as the measured distance. Then the distance error was calculated. The test results are illustrated in Figure 14.

Compared with the method in [42], the method proposed in this paper effectively reduced the error in the distance range of 0.5–5 m. Meanwhile, the adaptivity under different environments improved a lot. Table 4 gives the detailed performance comparison results of the two methods.

The maximal error, average error and RMSE were chosen to compare the detailed performance. From Table 4, the maximum error was reduced distinctly in the proposed method, while the non-uniformity reduced a lot, too. Though the difference of average error was not distinct as other two indicators, the proposed method had better adaptability. In other words, the proposed was more adaptive to multiscene and different reflectivities.

The proposed method was compared with several traditional methods as well, and the result is shown in Table 5. It can be obviously figured out that the proposed method has better performance on range accuracy compared with methods in [13,17,26]. Although the mean distance error shows no distinct improvement compared with method in [19], the proposed method has better performance on calibration time and scene scope. In addition, the results of experiments expressed that the proposed method had an outstanding performance on adaptability.

## 5. Conclusions

To improve the range accuracy of the PMD solid-state array Lidar, this paper presents a self-adaptive grayscale correlation based depth calibration method (SA-GCDCM) based on electrical analog delay. Based on the characteristic of the PMD solid-state array Lidar, the grayscale image was used to estimate the parameters of ambient light compensation in depth calibration. To obtain uniform and stable grayscale image, an integration time simulating based method was proposed for eliminating the influence of DSNU and PRNU. Combining SA-GCDCM and demodulation error correction, DRNU error compensation and temperature compensation, a comprehensive, multiscene adaptive multifactor calibration model was established. A series of experiments were conducted to verify the ranging accuracy and the adaptability of the method. Compared with the prior work, the maximum error has reduced distinctly, meanwhile the RMSE was reduced as well, indicating the proposed method had better accuracy and adaptability, respectively. Compared with the traditional methods, the proposed method had better performance on range accuracy and calibration time and scene scope. The proposed method was more adaptive to multiscenes with targets of different reflectivities, which significantly improved the ranging accuracy and adaptability of PMD Lidar.

## Figures and Tables

**Table 1:** Pixel adaptive interpolation strategy.

Case	Pixel Adaptive Interpolation Strategy
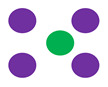	$\begin{array}{cc} D_{0}=(1-a_{x})\times & (1-a_{y})\times D_{1}+(1-a_{x})\times a_{y}\times D_{2}\\ & +a_{x}\times (1-a_{y})\times D_{3}+a_{x}\times a_{y}\times D_{4} \end{array}$
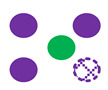	$D_{0}=uD_{1}+vD_{2}+\left(1-u-v\right)D_{4}$
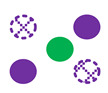	$D_{0}=\left(1-\frac{x_{p0}+y_{p0}}{2}\right)\times D_{1}+\frac{x_{p0}+y_{p0}}{2}\times D_{4}$
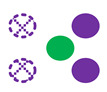	$D_{0}=a_{y}D_{4}+\left(1-a_{y}\right)\times D_{3}$
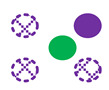	$D_{0}=D_{x}$
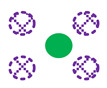	$D_{0}=NaN$

**Table 2:** Lens parameters.

**Internal Parameters**	***f_x_***	***f_y_***	***c_x_***	***c_y_***
	208.915	209.647	159.404	127.822
**Distortion Coefficients**	***k_1_***	***k_2_***	***p_1_***	***p_2_***
	−0.37917	0.17410	0.00021	0.00124

**Table 3:** Quantitative analysis of the grayscale calibration method.

	Flat White Board		Real Scene 1		Real Scene 2	
	Before	After	Before	After	Before	After
**Mean value**	913.98	915.73	559.85	571.83	278.91	305.03
**RMSE**	168.22	12.89	291.30	221.23	256.65	192.86
**PSNR**	43.97	55.13	41.58	42.78	42.13	43.37

**Table 4:** Detailed performance comparison results of the two methods.

Comparison Items	Maximal Error (mm)	Average Error (mm)	RMSE (mm)
The proposed method	16.4	8.13	4.47
Reference [42] method	20.5	9.68	5.56

**Table 5:** Detailed performance compared with traditional methods.

	Distance Error (mm)									Calibration Time	Scene Scope
	900	1100	1300	1700	2100	2500	3000	3500	4000		
Lindner et al. [13]	19.4	28.2	21.0	28.9	13.5	17.3	15.9	21.8	26.7	About dozens of minutes	About 4 m × 0.6 m × 0.4 m
Steiger et al. [17]	NaN	3(at 1207)		25	57	NaN	NaN	NaN	NaN	About dozens of minutes	Not mentioned
Schiller et al. [19] (Automatic feature detection)	7.45 (mean)							NaN	NaN	About dozens of minutes	About 3 m × 0.6 m × 0.4 m
Schiller et al. [19] (Some manual feature selection)	7.51 (mean)							NaN	NaN	About dozens of minutes	About 3 m × 0.6 m × 0.4 m
Huang et al. [26]	42	23	18	24	46	60	58	76	NaN	Not mentioned	About 1.5 m × 1.5 m × 2 m
The proposed method	3.1	4.4	5.5	7	7.4	8.1	9.8	9.6	12	90 s(calculation) 10 min (calculation, scene setup and initialization)	About 1.0 m × 1.0 m × 1.5 m
